# A prognostic signature based on three-genes expression in triple-negative breast tumours with residual disease

**DOI:** 10.1038/npjgenmed.2015.15

**Published:** 2016-02-03

**Authors:** Joseph A Pinto, Jhajaira Araujo, Nadezhda K Cardenas, Zaida Morante, Franco Doimi, Tatiana Vidaurre, Justin M Balko, Henry L Gomez

**Affiliations:** 1 Unidad de Investigación Básica y Traslacional, Oncosalud-AUNA, Lima, Peru; 2 Escuela de Medicina Humana, Universidad Privada San Juan Bautista, Lima, Peru; 3 Departamento de Patología, Instituto Nacional de Enfermedades Neoplásicas, Lima, Peru; 4 Departamento de Oncología Médica, Instituto Nacional de Enfermedades Neoplásicas, Lima, Peru; 5 Department of Medicine, Vanderbilt-Ingram Comprehensive Cancer Center, Vanderbilt University, Nashville, TN, USA

## Abstract

Residual disease after neoadjuvant chemotherapy (NAC) in triple-negative breast cancer (TNBC) is related with poor prognosis; however, the risk of recurrence after 3 years from surgery, becomes similar to other breast cancer subtypes indicating that TNBC is composed of tumours of different prognosis. To evaluate genes related to TNBC aggressiveness in the outcome of TNBC resistant to NAC, we profiled 82 samples of residual tumours whose expression for 449 genes was quantified with NanoString. The validation set (GSE25066) consisted of 113 TNBC cases with residual disease. The stepwise multivariate survival analysis performed by the Cox proportional hazards mode selected *CCL5, DDIT4* and *POLR1C* as independent prognostic factors for distant recurrence-free survival (DRFS). We developed a three-genes signature using the regression coefficients for each gene (−0.393×*CCL5*+0.443×*DDIT4*+0.490×*POLR1C*). The median score in the discovery set (0.1494) identified two subgroups with different DRFS (*P*<0.001). The median score in the validation set was 0.0024 and was able to discriminate patients with different DRFS (*P*=0.002). In addition, the three-genes signature was a prognostic factor in TNBC patients regardless their response to NAC (data set GSE58812; *P*=0.001) and in patients with oestrogen-receptor-negative tumours (data set GSE16446; *P*=0.041). Here we describe a prognostic signature based on expression levels of *CCL5, DDIT4* and *POLR1C*. The knowledge about the involvement of these genes in chemotherapy resistance could improve the therapeutic strategies in TNBC.

## Introduction

The triple-negative breast cancer (TNBC) is the most aggressive subtype of breast tumours due to limited therapeutic options using targeted drugs and is biologically characterized by absence of expression of oestrogen receptor (ER), progesterone receptor (PR), and HER2 receptor.^1^ According to its molecular characteristics, TNBC could be classified the in six different subtypes.^2^

Race/ethnicity is a factor related to TNBC incidence, as these tumours are more frequent in African-Americans (21%) than in Caucasians (9–15%).^3^ TNBC incidence has been described in several Latin America countries with a 21.3% frequency in Peru, 23.1% in Mexico, 24.6% in Venezuela and 27% in Brazil.^4–7^ Considering breast cancer incidence in Peru (28/100,000 women), 850 out of ≈4,000 cases diagnosed each year would correspond to TNBC.^4,8^

The pathological complete response (pCR) to neoadjuvant chemotherapy (NAC) is the best predictor of distant recurrence-free survival (DRFS) and overall survival; however, 3 years after surgery, the risk of recurrence and death is similar than other breast cancer subtypes, indicating that TNBC is composed by a mix of tumours with different prognosis.^9^ This observation could be explained by the molecular heterogeneity of TNBC, composed by subtypes with different clinical outcomes, where the basal type 1 achieve the highest pCR rates (52%), while the luminal-androgen receptor and the basal-like 2 subtypes achieve lower response rates (10% and 0%, respectively).^10^

Nowadays, there are commercially avalaible tests based on levels of gene expression; such as, the 21-genes recurrence score (OncotypeDx) and the 70-genes signature (Mammaprint). Despite the clinical utility, these tests have certain limitations. The 21-genes score (16 cancer-related and 5 control genes) calculates a recurrence score between 0 and 100, where the risk of recurrence increase with the score and estimate the likelihood of benefit with the adjuvant chemotherapy. This test is recommended by NCCN guidelines only in patients diagnosed with pT1–pT3 and pN0 and pN1mi ER+/HER2− breast tumours.^11–13^ On the other hand, the 70-genes signature calculates a score to assign a risk group (high or low risk) and estimate the probability of 10 years recurrence from diagnosis.^14^ The 70-genes score (although not recommended by NCCN guidelines) has FDA approval for luminal patients with stage I or II, with ⩽3 lymph nodes involved and invasive tumours <5 cm. Despite these molecular platforms have shown clinical utility, these genomic predictors are not useful in TNBC and new markers and predictors are needed in order to a improve the risk stratifications and therapeutic strategies.

The aim of our study was to evaluate genes related to TNBC aggressiveness to elaborate a gene signature associated with prognosis in terms of DRFS in TNBC resistant to NAC.

## Results

### Selection of genes related with DRFS

**Figure 1:**
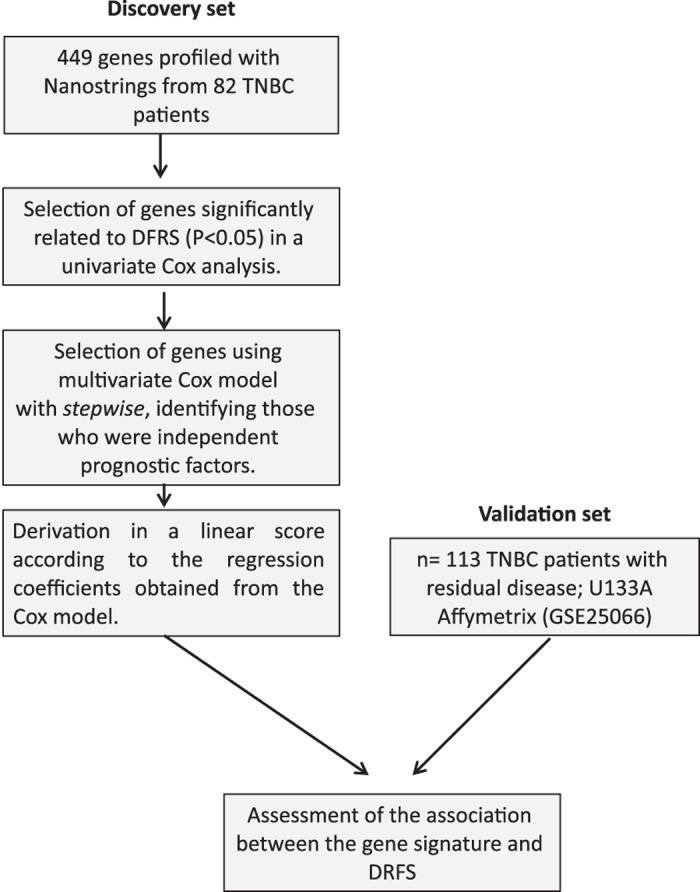
Overview of research design and workflow.

Workflow is shown in Figure 1. Overall, median DRFS in the discovery set was 22.3 months. Univariate Cox regression of 449 genes related to aggressiveness signatures identified 7 genes statistically related to DRFS (*P*<0.05): *CCL5*, *CYBB*, *DDIT4*, *GTPBP4*, *KRT6B*, *PALMD* and *POLR1C*.

### Three-genes prognostic score

Stepwise multivariate survival analysis performed by the Cox proportional hazards model of the seven genes from the previous step, selected three genes as independent prognostic factors: *CCL5* (Chemokine (CC motif) ligand 5), *DDIT4* (DNA-damage-inducible genes transcript 4) and *POLR1C* (Polymerase (RNA] I polypeptide C, 30 Kd1a)*,* with *P*-values of 0.002, 0.005 and 0.004, respectively. CCL5 had protective effect (hazard ratio (HR)<1), whereas *DDIT4* and *POLR1C* were associated to a worse outcome (HR>1; Table 1).

The three-genes prognostic score results from the sum of multiplication of normalised expression levels of the genes by their respective regression coefficient as is described in the following formula:

Three-genesprognosticscore=(−0.393×CCL5+0.443×DDIT4+0.490×POLR1C)

The median score in the discovery set was 0.1494. Using this median as cutoff, was possible to establish two risk groups.

### Individual value of *CCL5, DDIT4* and *POLR1C* in TNBC

**Figure 2:**
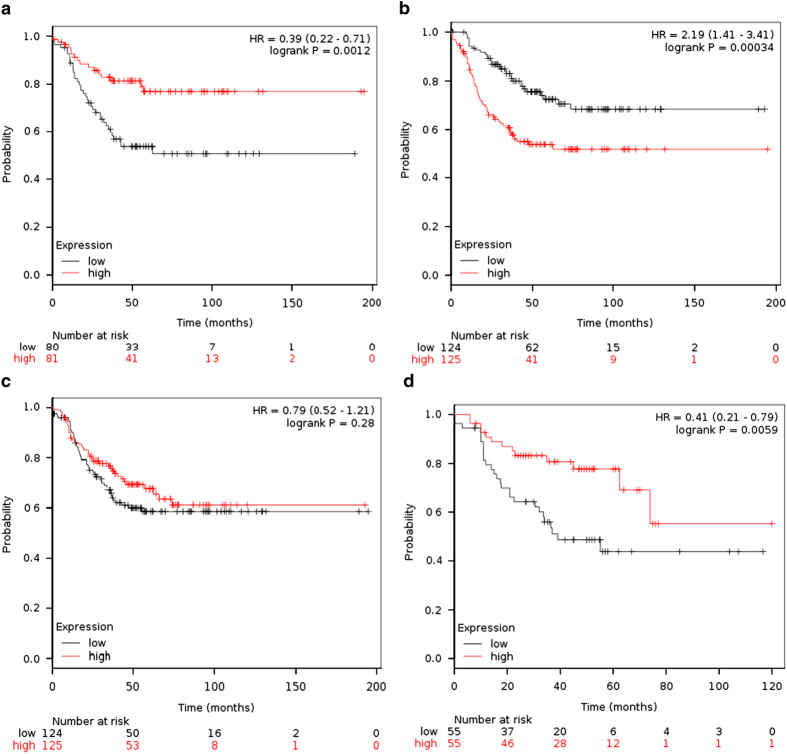
Individual impact of *CCL5, DDIT4* and *POLR1C* genes recurrence-free survival (RFS) in triple-negative breast cancer patients (using the median of expression as cutoff) in the database of the online platform kmplot.org. (**a**) High expression of *CCL5* was associated with good prognosis (*P*=0.0012). (**b**) High expression of *DDIT4* confers poor prognosis (*P*=0.00034). (**c**) Expression of *POLR1C* was not significantly associated with the RFS in all patients (*P*=0.28); however, (**d**) excluding systematically untreated patients, a significant association is observed (0.0059).

We evaluated *CCL5, DDIT4* and *POLR1C* in the Kaplan–Meier Plotter online platform (kmplot.org) in order to analyse their influence in the recurrence-free survival (RFS) of TNBC patients.^15^ When group of patients was split into two groups according to median of expression, *CCL5* and *DDIT4* were associated with RFS (*P*=0.0012 and *P*=0.00034, respectively). *POLR1C* was not statistically associated with RFS (*P*=0.28); however, when systematically untreated patients were removed from the cohort, high expression of *POLR1C* was associated with a poor prognosis (*P*=0.0059; Figure 2).

### Prognostic value of the three-genes signature

**Figure 3:**
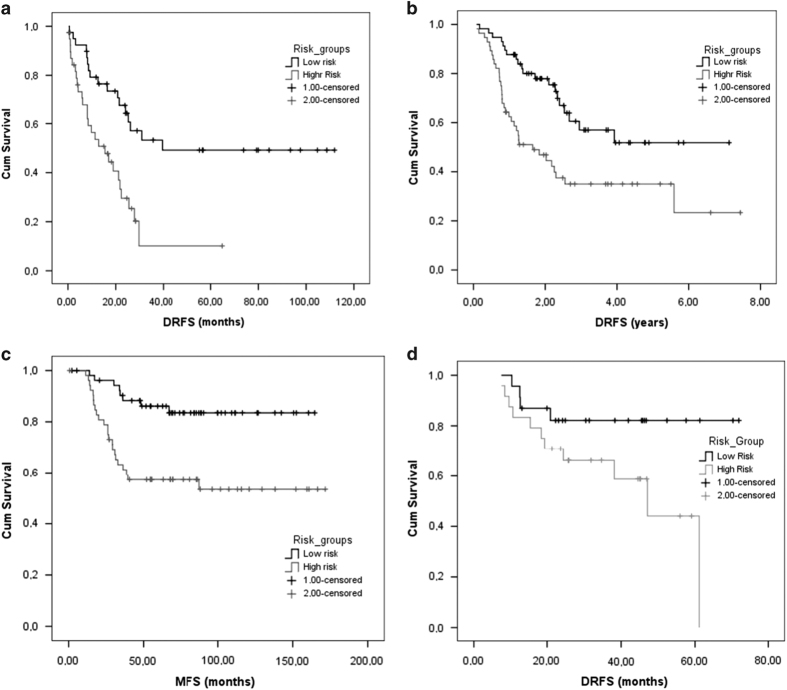
Three-genes score dichotomised at the median as prognostic factor in terms of DRFS and MFS. (**a**) Survival curves of 82 TNBC in the discovery set (*P*<0.001); (**b**) 113 patients in the validation set (*P*=0.002); (**c**) in 107 TNBC patients regardless their response to neoadjuvant chemotherapy and (*P*=0.001) and (**d**) 47 patients with ER(−) and HER2 non-amplified breast tumours (*P*=0.041).

Evaluating the score as a continuous variable in the CoxPH analysis, a HR=2.72 for each unit change (*P*<0.001; 95% CI: 1.72–4.28) was estimated. The risk groups established by the median cutoff shown statistically significant differences in the DRFS, with a median survival of 39.6 months for the low-risk group and 15.5 months for the high-risk group (*P*<0.001; Figure 3a).

### Validation cohort

#### Validation set (GSE25066)

The median DRFS in this group of patients was 35.4 months. In this group, the median of the score was 0.0024 and was able to identify two subgroups with different outcomes where the median DRFS for the low-risk group was not reached and the median DRFS for the high-risk group was 22 months (*P*=0.002) (Figure 3b).

### Analysis of other data sets

#### Patients with TNBC regardless of their response to NAC (GSE58812)

The end point evaluated in this data set was metastases-free survival (MFS). The median score was −0.1009. The median of MFS was not reached and 4-year MFS rates were 83.5% vs 57.3% for low-risk vs high-risk groups according the three-genes signature (*P*=0.001; Figure 3c).

#### Patients with oestrogen negative tumours (GSE16446)

The median DRFS was 61.1 months. The median score was 0.0137 and was able to classify into two subgroups with different prognosis, where the median DFRS for the low-risk group was not reached and the median DRFS for the high-risk group was 47.1 months (*P*=0.041; Figure 3d).

### Multivariate analysis

In the discovery set, in addition to three-genes prognostic signature, age, lymph node status were statistically related to the DFRS in the univariate analysis. In the multivariate analysis, only >3 involved nodes (HR=3.98, 95% CI: 1.73–9.12) and a poor three-genes prognostic signature (HR=2.03, 95% CI: 1.02–4.05) were independent factors associated with shorter DFRS (Table 2). In the validation set, in addition to the three-genes prognostic signature, the T-stage, nodal stage and clinical AJCC stage were statistically related to DFRS. In the multivariate analysis, T3–4 stage (HR=1.878; 95% CI: 1.054–3.346), nodal stage 2–3 (HR=2.78, 95% CI: 1.271–6.079) and the high-score group (HR=2.358, 95% CI: 1.359–4.091) were independent poor prognostic factors (Table 3).

## Discussion

Triple-negative breast cancer is a classification obtained by exclusion criteria rather than representing a well-defined entity such as other breast cancer subtypes.^16^ The pCR after NAC is the main indicator for good prognosis. In Peru, a study by Neciosup *et al.*,^17^ reported pCR rates of 9%, lower frequency than those reported in other series for TNBC (≈22% and up to 36% with addition of bevacizumab in the neoadjuvant setting).^10,17,18^

In the last decade, there has been growing interest to unveil the molecular biology of TNBC leading to identification of six TNBC subtypes with distinct biology and prognosis, where our research group could identify loss of *DUSP4* expression as mechanism of resistance to NAC and 90% of TNBC have an actionable mutation candidates to be treated with targeted drugs.^19,20^

In our study we found three genes independently related with the outcome in several TNBC data sets. In addition, these genes can be combined in a linear score. Unfortunately, there is unavailability of certain clinical data in public data sets, including time-to-event data, leading to an important limitation in its use. Lack of important information in public data sets despite journal requirements has been previously addressed.^21^ To overcome this issue, we selected patients with residual disease from data sets profiled with a different platform (affymetrix microarrays) and with samples taken prior NAC for our validation cohort. Although it is expected that gene expression patterns could change after NAC, our three-genes signature was able to identify groups with different prognosis evaluating samples profiled before NAC. On the other hand, *CCL5, DDIT4* and *POLR1C* in samples profiled after treatment in large data sets of TNBC patients (evaluated in the online platform kmplot.org) shown a significant association of gene expression levels with the RFS (Figure 2).

The gene *CCL5* is located on 17q, and is part of a superfamily of secreted proteins involved in immunoregulatory and inflammatory processes. TCGA (The Cancer Genome Atlas) data show that 4% of breast tumours have *CCL5* dysregulation (2% in basal tumours), mainly related to genetic downregulation which has been previously associated with breast cancer progression.^22^ *CCL5* expressed by tumours recruits tumour infiltrant lymphocytes (TILs). TILs has an important role in the outcome of TNBC where >20% of TILs are associated with an better prognosis, while lower proportion of TILs (<10%) is associated with genetic or transcriptomic alterations in Ras/MAPK pathway.^23^ On the other hand, *CCL5* was shown to be a mechanism of tumour escape in a mouse model of colorectal cancer, recruiting and improving the cytotoxic effects of T-regulatory cells against CD8+ T cells.^24^

The *DDIT4* gene (DNA-damage-inducible transcript 4) encodes a protein related to adverse environmental conditions, whose action is the inhibition of mTOR.^25^ Despite the biological function of *DDIT4*, in our analysis this gene was related with tumour aggressiveness with an HR=1.56 (*P*=0.005) by each unit of change (Table 1). A recent report by Puissant *et al.*,^26^ describe that the product of *DDIT4* could be cleaved by caspase 3 modifying its cellular function exerting anti-proliferative activities.^26^ TCGA data show that dysregulation of this gene in 4% of breast tumours, mainly overexpression (altered in 12% of tumours basal subtype).

The *POLR1C* gene (30 kDa), encodes a subunit of the RNA polymerase enzymes I and III, responsible for RNA synthesis.^27^ The mutation in this gene causes Treacher Collins syndrome that affects the development of bones and facial tissues.^28^ *POLR1C* gene is recurrently amplified and overexpressed in gastric cancer.^29^ TCGA data indicate that 11% of breast cancer cases have dysregulations in this gene (overexpression and downregulation). This gene is dysregulated in 21% of cases of basal tumours, where changes include overexpression. The molecular mechanisms of its involvement in tumour aggressiveness remain unclear.

In spite that this cohort of TNBC patients was evaluated previously and other genes were found to be related significantly with the outcome, such as a signature of MEK pathway activation and *DUSP4* loss (corroborated *in vivo* and *in vitro* experiments), in the analysis we done in this work found other genes not previously reported.^19,30^ It is important to explore biomarkers under different strategies, because weak biological signals or interaction between markers could be ignored in some bioinformatics or statistical analysis, for example, when is used as multiple testing procedures.^31–33^

Owing to the TNBC heterogeneity, some cases of this phenotype could correspond to luminal A, luminal B or HER2-enriched subtypes. In TNBC, the PAM50 test has a particular utility. PAM50 evaluates 50 genes required for determining intrinsic molecular subtypes of breast cancer.^34^ Because immunohistochemistry has limited ability to detect protein expression, PAM50 becomes a test that can identify more accurately the molecular subtypes within the TNBC phenotype, providing additional information for deciding a better therapeutic strategy.^35,36^

Our prognostic signature, unlike other commercially available platforms, is based only in three-genes expression. This finding could lead to the development of a cheap molecular test based in reverse transcription PCR (RT-PCR) and suitable in the clinical routine. The different median values of the score obtained in each data set could be explained by the different prognostic factor between them and/or differences between microarray platforms. On the another hand, our three-genes prognostic score produced robust results and could be evaluated in both biopsies and residual tumours after NAC.

In conclusion, our analysis of 449 genes related to aggressive molecular signatures identified three genes (*CCL5*, *DDIT4* and *POLR1C*) that were independent prognostic factors whose combination resulted in a predictor able to identify TNBC patients with different outcome. These data encourage the prospective clinical validation of these genes using the technology of real-time PCR. The products of *CCL5*, *DDIT4* and *POLR1C* genes could be used for develop new therapeutic strategies in TNBC and basal breast cancer.

## Materials and methods

### Discovery set

We evaluated 114 patients with TNBC who had residual tumour after NAC (diagnosed and treated at the Instituto Nacional de Enfermedades Neoplásicas, Lima, Peru). Cases with insufficient tissue or cellularity (*n*=5) or HER2-amplified (*n*=7) were excluded. In total, 82 cases were evaluable for analysis, Formalin-fixed paraffin-embedded residual tumour from surgical specimens were serially cut into 3- to 5-μm thick, then cellularity evaluation and nucleic acid extraction was done. RNA was extracted and purified using the RNEasy FFPE kits (Qiagen GmbH, Hilden, Germany).

### Genes selected for evaluation

Overall, evaluation of 449 transcripts were selected based on their inclusion in published gene expression signatures: the *PAM50* genes, signatures associated with *DUSP4* loss, with MEK activation, with the enrichment of TGFβ inducible genes after NAC or signatures based on their association with the post-NAC Ki-67 score.^19,34,37,38^

### NanoString nCounter analysis

NanoString nCounter technique captures and counts individual messenger RNA transcripts using unique pairs of capture and reporter probes with a colour code generated by ordered fluorescent segments specific for each transcript. As enzymatic reactions are not used, there is no bias or decrease of sensitivity compared with other techniques, such as microarrays.^39^ RNA extracted from formalin-fixed paraffin-embedded residual tumour was run on the nCounter Analysis System (NanoString Technologies, Seattle, WA, USA), according to the manufacturer’s protocol. Code sets were synthesised targeting 499 genes.

### Validation set

#### GSE25066

We selected 113 TNBC (determined by immunohistochemistry) with residual disease after NAC. Gene profiling was done with U133A Affymetrix microarray platform (Affymetrix U133A chip, Santa Clara, CA, USA) in biopsies before NAC. List microarray probes evaluated in this data set are shown in Supplementary Table S1.

### Analysis of other data sets

#### GSE58812

Composed by 107 TNBC patients regardless their response to neoadjuvant treatment (these data were not included in the data set. U133A Affymetrix microarray platform in biopsies before NAC. List of microarray probes evaluated in this data set are shown in Supplementary Table S1.

#### GSE16446

All patients in this data set were ER negative. We selected 47 patients with HER2 not-amplified tumours. Gene profiling with the U133 Plus 2.0 Affymetrix array was done in biopsies before NAC. List of microarray probes evaluated in this data set are shown in Supplementary Table S2.

### Gene expression data preprocessing

In the discovery set, expression levels were normalised with Spike-controls*,* log_2_ transformed and median centred. In the data sets GSE25066, GSE58812 and GSE16446, probes for the same gene were collapsed to the higher value, then the expression data were log_2_ transformed and median centred. Values of normalised data values are shown in Supplementary Tables S3–S6.

### Prognostic signature

The expression of 449 genes was evaluated to select genes strongly associated to DRFS (*P*<0.05) using Cox regression models. Genes significantly associated with DFRS were further tested using Cox proportional hazards regression with the stepwise method of selection, identifying those genes that were independent prognostic factors. We used a linear combination of the normalised values of gene expression levels multiplied by a weighting value for each gene (regression coefficients) to calculate a risk score for each patient. The proportional hazards assumption over time for the final Cox model for the dichotomised risk score was tested graphically using log–log survival functions, and the assumption of appropriateness in discovery and validation sets was confirmed (Supplementary Figure S1).

### Survival analysis

DRFS was estimated by the Kaplan–Meier method. The log-rank test was used as the method of statistical inference. After calculating the risk score, the median was estimated. Using the median (specific to each group of patients) as a cutoff, two risk subgroups were established. Survival curves were compared using the log-rank test. A *P* value <0.05 was considered statistically significant.

### Ethical considerations

This study involves a reanalysis of gene expression and clinical data obtained in a previous study that was approved by the IRB from the Instituto Nacional de Enfermedades Neoplasicas (INEN 10–018).

## Figures and Tables

**Table 1:** Genes selected as independent prognostic factors in the stepwise multivariate survival analysis performed by the Cox proportional hazards model in the discovery set

*Gene*	*Discovery set*	P-value	*Regression coeficient*
	*HR (95% CI)*		
*CCL5*	0.68 (0.53–0.86)	0.002	−0.393
*DDIT4*	1.56 (1.14–0.76)	0.005	0.443
*POLR1C*	1.63 (1.17–2.28)	0.004	0.490

Abbreviations: CI, confidence interval; HR, hazard ratio.

Values of hazard ratios and regression coefficients are shown.

**Table 2:** Univariate and multivariate analysis of patientś and tumour characteristics related to DRFS in the discovery set

	n *(events)*	*Univariate analysis*		*Multivariate analysis*	
		*HR (*95% CI*)*	P*-value*	*HR (*95% CI*)*	P*-value*
*Discovery cohort*					
Age			**0.010**		0.424
⩽45	30 (21)	1		1	
>45	49 (23)	0.46 (0.25–0.83)		0.68 (0.26–1–75)	
					
Menopausal status			**0.044**		0.544
Premenopausal	36 (24)	1		1	
Postmenopausal	43 (20)	0.54 (0.30–0.98)		0.75 (0.30–1.90)	
					
Stage			0.127		
IIA, IIB	6 (5)	1			
IIIA, IIIB, IIIC	73 (39)	0.48 (0.19–1.23)			
					
Involved lymph nodes			**0.003**		**0.026**
0	25 (9)	1		1	
1–3	31 (18)	1.66 (0.75–3.70)	0.213	1.37 (0.60–3.14)	0.450
>3	22 (16)	3.98 (1.73–9.12)	**0.001**	3.98 (1.73–9.12)	**0.014**
					
Ki-67 score			0.192		
⩽14	15 (10)	1			
>14	64 (34)	0.62 (0.31–1.27)			
					
Prognostic signature			**0.001**		**0.044**
Low risk	40 (17)	1		1	
High risk	39 (27)	2.86 (1.52–5.36)		2.03 (1.02–4.05)	

Abbreviations: CI, confidence interval; HR, hazard ratio.Bold entries denote statistically significant values.

**Table 3:** Univariate and multivariate analysis of patientś and tumour characteristics related to DRFS in the validation set 1 (GSE25066)

	n *(events)*	*Univariate analysis*		*Multivariate analysis*	
		*HR (95% CI)*	P*-value*	*HR (95% CI)*	P*-value*
*GSE25066 validation set*					
Age			0.656		**0.032**
⩽45	44 (20)	1			
>45	69 (35)	0.656 (0.508–1.532)			
					
Clinical T stage			**0.006**		
0–2	52 (18)	1		1	0.032
3–4	61 (37)	2.172 (1,232–3,831)		1.878 (1.054–3.346)	
					
Clinical nodal stage			**0.010**		0.025
0	26 (9)	1		1	
1	52 (23)	1.706 (0.785–3.711)	0.178	1.609 (0.740–3.499)	
2–3	35 (23)	3.109 (1.429–6.767)	**0.004**	2.779 (1.271–6.079)	
					
Clinical AJCC stage			**0.004**		
I–IIB	51 (18)	1			
IIIA, IIIB, IIIC, Inflammatory	62 (37)	2.3 (1,306–4,048)			
					
Histological grade			0.542		
2	17 (9)	1			
3	86 (38)	0.798 (0.386–1.651)			
					
Prognostic signature			**0.002**		**0.003**
Low risk	57 (20)	1		1	
High risk	56 (35)	2.358 (1.359–4.091)		2.308 (1.326–4.018)	

Abbreviations: CI, confidence interval; HR, hazard ratio. Bold entries denote statistically significant values.

## References

[bib1] Dent, R. et al. Triple-negative breast cancer: clinical features and patterns of recurrence. Clin. Cancer Res. 13, 4429–4434 (2007). 17671126 10.1158/1078-0432.CCR-06-3045

[bib2] Lehmann, B. D. et al. Identification of human triple-negative breast cancer subtypes and preclinical models for selection of targeted therapies. J. Clin. Invest. 121, 2750–2767 (2011). 21633166 10.1172/JCI45014 PMC3127435

[bib3] Carey, L. A. et al. Race, breast cancer subtypes, and survival in the Carolina Breast Cancer Study. JAMA 295, 2492–2502 (2006). 16757721 10.1001/jama.295.21.2492

[bib4] Vallejos, C. S. et al. Breast cancer classification according to immunohistochemistry markers: subtypes and association with clinicopathologic variables in a Peruvian hospital database. Clin. Breast Cancer 10, 294–300 (2010). 20705562 10.3816/CBC.2010.n.038

[bib5] Lara-Medina, F. et al. Triple-negative breast cancer in Hispanic patients: high prevalence, poor prognosis, and association with menopausal status, body mass index, and parity. Cancer 117, 3658–3669 (2011). 21387260 10.1002/cncr.25961

[bib6] Márquez, M., Lacruz, J. C. & Borges, L. F. Sobrevida en pacientes con cancer de mama triple negativo. Rev. Obstet Ginecol. Venez. 72, 152–160 (2012).

[bib7] Amaral, A. L., Vitral, I. & Koifman, S. Triple negative breast cancer in Brazilian women without metastasis to axillary lymph nodes: ten-year survival and prognostic factors. Br. J. Med. Med. Res. 3, 880–896 (2013).

[bib8] Ferlay J. et al. GLOBOCAN 2012 v1.0, Cancer Incidence and Mortality Worldwide: IARC CancerBase No. 11. International Agency for Research on Cancer. Avalaible at http://globocan.iarc.fr. Accessed on 12 March 2015.

[bib9] Liedtke C. et al. Response to neoadjuvant therapy and long term survival in patients with triple negative breast cancer. J. Clin. Oncol. 2008; 26: 1275–1281. 18250347 10.1200/JCO.2007.14.4147

[bib10] Liedtke, C. et al. Differential response to neoadjuvant chemotherapy among 7 triple-negative breast cancer molecular subtypes. Clin. Cancer Res. 19, 5533–5540 (2013). 23948975 10.1158/1078-0432.CCR-13-0799 PMC3813597

[bib11] Paik, S. et al. A multigene assay to predict recurrence of tamoxifen-treated, node-negative breast cancer. N. Engl. J. Med. 351, 2817–2826 (2004). 15591335 10.1056/NEJMoa041588

[bib12] Paik, S. et al. Gene expression and benefit of chemotherapy in women with node-negative, estrogen receptor-positive breast cancer. J. Clin. Oncol. 24, 3726–3734 (2006). 16720680 10.1200/JCO.2005.04.7985

[bib13] National Comprehensive Cancer Network. Clinical Practice Guidelines in Oncology (NNCN guidelines). Breast Cancer V.3. 2015. Avalaible at http://www.nccn.org/professionals/physician_gls/pdf/breast.pdf. Accessed on 15 April 2015.

[bib14] Buyse, M. et al. Validation and clinical utility of a 70-gene prognostic signature for women with node-negative breast cancer. J. Natl. Cancer. Inst. 98, 1183–1192 (2006). 16954471 10.1093/jnci/djj329

[bib15] Györffy, B. et al. An online survival analysis tool to rapidly assess the effect of 22,277 genes on breast cancer prognosis using microarray data of 1,809 patients. Breast Cancer Res. Treat. 123, 725–731 (2010). 20020197 10.1007/s10549-009-0674-9

[bib16] Perou, C. M. Molecular stratification of triple-negative breast cancers. Oncologist 15S, 39–48 (2010). 10.1634/theoncologist.2010-S5-39 21138954

[bib17] Neciosup, S., Marcelo, M., Venrtura, L., Vallejos, C. & Gomez, H. Factores asociados a la respuesta patológica a la quimioterapia en el cáncer de mama triple negativo. Carcinos 1, 11–17 (2012).

[bib18] Gerber, B. et al. Neoadjuvant bevacizumab and anthracycline-taxane-based chemotherapy in 678 triple-negative primary breast cancers; results from the geparquinto study (GBG 44). Ann. Oncol. 24, 2978–2984 (2013). 24136883 10.1093/annonc/mdt361

[bib19] Balko, J. M. et al. Profiling of residual breast cancers after neoadjuvant chemotherapy identifies DUSP4 deficiency as a mechanism of drug resistance. Nat. Med. 18, 1052–1059 (2012). 22683778 10.1038/nm.2795 PMC3693569

[bib20] Balko, J. M. et al. Molecular profiling of the residual disease of triple-negative breast cancers after neoadjuvant chemotherapy identifies actionable therapeutic targets. Cancer Discov. 4, 232–245 (2014). 24356096 10.1158/2159-8290.CD-13-0286 PMC3946308

[bib21] Alsheikh-Ali, A. A., Qureshi, W., Al-Mallah, M. H. & Ioannidis, J. P. Public availability of published research data in high-impact journals. PLoS ONE 6, e2435 (2011). 10.1371/journal.pone.0024357 PMC3168487 21915316

[bib22] Soria, G. & Ben-Baruch, A. The inflammatory chemokines CCL2 and CCL5 in breast cancer. Cancer Lett. 267, 271–285 (2008). 18439751 10.1016/j.canlet.2008.03.018

[bib23] Loi S. et al. RAS/MAPK activation is associated with reduced tumour-infiltrating lymphocytes in triple-negative breast cancer: therapeutic cooperation between MEK and PD-1/PD-L1 immune checkpoint inhibitors. Clin. Cancer Res. (in press). 10.1158/1078-0432.CCR-15-1125 PMC4794351 26515496

[bib24] Chang, L. Y. et al. Tumour-derived chemokine CCL5 enhances TGF-β-mediated killing of CD8(+) T cells in colon cancer by T-regulatory cells. Cancer Res. 72, 1092–1102 (2012). 22282655 10.1158/0008-5472.CAN-11-2493

[bib25] Tan, C. Y. & Hagen, T. mTORC1 dependent regulation of REDD1 protein stability. PLoS ONE 8, e63970 (2013). 23717519 10.1371/journal.pone.0063970 PMC3661664

[bib26] Puissant, A. et al. A new posttranslational regulation of REDD1/DDIT4 through cleavage by caspase 3 modifies its cellular function. Cell Death Dis. 5, e1349 (2014). 25058423 10.1038/cddis.2014.291 PMC4123085

[bib27] Dammann, R. & Pfeifer, G. P. Cloning and characterization of the human RNA polymerase I subunit hRPA40. Biochim. Biophys. Acta 1396, 153–157 (1998). 9540830 10.1016/s0167-4781(97)00206-6

[bib28] Kadakia, S., Helman, S. N., Badhey, A. K., Saman, M. & Ducic, Y. Treacher Collins Syndrome: the genetics of a craniofacial disease. Int. J. Pediatr. Otorhinolaryngol. 78, 893–898 (2014). 24690222 10.1016/j.ijporl.2014.03.006

[bib29] Cheng, L. et al. Identification of genes with a correlation between copy number and expression in gastric cancer. BMC Med. Genomics 5, 14 (2012). 22559327 10.1186/1755-8794-5-14 PMC3441862

[bib30] Balko, J. M. et al. A gene expression signature of MEK pathway activation to predict survival in triple-negative breast cancer. J. Clin. Oncol. 30, (2012) (suppl; abstr 1024).

[bib31] Khoury, M. J., Little, J., Gwinn, M. & Ioannidis, J. P. On the synthesis and interpretation of consistent but weak gene-disease associations in the era of genome-wide association studies. Int. J. Epidemiol. 36, 439–445 (2007). 17182636 10.1093/ije/dyl253

[bib32] Saeys, Y., Inza, I. & Larrañaga, P. A review of feature selection techniques in bioinformatics. Bioinformatics 23, 2507–2517 (2007). 17720704 10.1093/bioinformatics/btm344

[bib33] Bickel, P. J., Brown, J. B. & Huang, H. An overview of recent developments in genomics and associated statistical methods. Phil. Trans. R. Soc. A 367, 4313–4337 (2009). 19805447 10.1098/rsta.2009.0164

[bib34] Parker, J. S. et al. Supervised risk predictor of breast cancer based on intrinsic subtypes. J. Clin. Oncol. 27, 1160–1167 (2009). 19204204 10.1200/JCO.2008.18.1370 PMC2667820

[bib35] Bastien, R. R. et al. PAM50 breast cancer subtyping by RT-qPCR and concordance with standard clinical molecular markers. BMC Med. Genomics 5, 44 (2012). 23035882 10.1186/1755-8794-5-44 PMC3487945

[bib36] Prat, A. et al. Predicting response and survival in chemotherapy-treated triple-negative breast cancer. Br. J. Cancer 111, 1532–1541 (2014). 25101563 10.1038/bjc.2014.444 PMC4200088

[bib37] Pratilas, C. A. et al. (V600E)BRAF is associated with disabled feedback inhibition of RAF-MEK signaling and elevated transcriptional output of the pathway. Proc. Natl Acad. Sci. USA 106, 4519–4524 (2009). 19251651 10.1073/pnas.0900780106 PMC2649208

[bib38] Bhola, N. E. et al. TGF-beta inhibition enhances chemotherapy action against triple-negative breast cancer. J. Clin. Invest. 123, 1348–1358 (2013). 23391723 10.1172/JCI65416 PMC3582135

[bib39] Geiss, G. K. et al. Direct multiplexed measurement of gene expression with color-coded probe pairs. Nat. Biotechnol. 26, 317–325 (2008). 18278033 10.1038/nbt1385

