# Supplementary material for: A prognostic signature based on three-genes expression in triple-negative breast tumours with residual disease
Source: NPJ Genom Med. 2016 Feb 3;1:15015–. doi: 10.1038/npjgenmed.2015.15 (PMC5685288; doi:10.1038/npjgenmed.2015.15)
Supplement: Supplementary Figures [file npjgenmed201515-s1.doc]

A)

B)

D)

C)

C)

Figure S1.- The proportional hazards assumption over time for appropriateness of the prognostic value of dichotomized risk score tested graphically using log-log survival functions for (A) the discovery set and datasets (B) GSE25066, (C) GSE58812 and (D) GSE16446.
